# Automated 3-dimensional MRI segmentation for the posterosuperior rotator cuff tear lesion using deep learning algorithm

**DOI:** 10.1371/journal.pone.0284111

**Published:** 2023-05-18

**Authors:** Su Hyun Lee, JiHwan Lee, Kyung-Soo Oh, Jong Pil Yoon, Anna Seo, YoungJin Jeong, Seok Won Chung

**Affiliations:** 1 Department of Orthopaedic Surgery, Seoul Red Cross Hospital, Seoul, Korea; 2 Department of Orthopedic Surgery, Myongji Hospital, Goyang-si, Korea; 3 Department of Orthopaedic Surgery, Konkuk University School of Medicine, Seoul, Korea; 4 Department of Orthopaedic Surgery, Kyungpook National University College of Medicine, Daegu, Korea; 5 SEEANN Solution, Yeonsu-gu, Incheon, Korea; University of Wisconsin-Eau Claire, UNITED STATES

## Abstract

**Introduction:**

Rotator cuff tear (RCT) is a challenging and common musculoskeletal disease. Magnetic resonance imaging (MRI) is a commonly used diagnostic modality for RCT, but the interpretation of the results is tedious and has some reliability issues. In this study, we aimed to evaluate the accuracy and efficacy of the 3-dimensional (3D) MRI segmentation for RCT using a deep learning algorithm.

**Methods:**

A 3D U-Net convolutional neural network (CNN) was developed to detect, segment, and visualize RCT lesions in 3D, using MRI data from 303 patients with RCTs. The RCT lesions were labeled by two shoulder specialists in the entire MR image using in-house developed software. The MRI-based 3D U-Net CNN was trained after the augmentation of a training dataset and tested using randomly selected test data (training: validation: test data ratio was 6:2:2). The segmented RCT lesion was visualized in a three-dimensional reconstructed image, and the performance of the 3D U-Net CNN was evaluated using the Dice coefficient, sensitivity, specificity, precision, F1-score, and Youden index.

**Results:**

A deep learning algorithm using a 3D U-Net CNN successfully detected, segmented, and visualized the area of RCT in 3D. The model’s performance reached a 94.3% of Dice coefficient score, 97.1% of sensitivity, 95.0% of specificity, 84.9% of precision, 90.5% of F1-score, and Youden index of 91.8%.

**Conclusion:**

The proposed model for 3D segmentation of RCT lesions using MRI data showed overall high accuracy and successful 3D visualization. Further studies are necessary to determine the feasibility of its clinical application and whether its use could improve care and outcomes.

## Introduction

Rotator cuff tear (RCT) is one of the most common diseases in the orthopedic field [1] with a reported prevalence of 9.7% to 62% [2, 3]. For the diagnosis of RCT, MRI is commonly used [4, 5], as it provides a lot of information about RCT, such as tear size and location, fatty infiltration, and other concomitant shoulder pathologies [6, 7]. However, an accurate interpretation of MRI for the diagnosis of RCT is difficult for non-musculoskeletal radiologists or non-orthopedic surgeons. It is also a time-consuming and tedious process that requires reviewing many MRI sequences.

Meanwhile, along with the development of artificial intelligence (AI), the technology to deal with clinical data has shown significant success in the medical industry [8]. Recently, deep learning methods to analyze medical images have been introduced in various medical fields, such as skin cancer [9], diabetic retinopathy [10], and lung nodules [11]. More recently, medical image analysis using deep learning methods has been extended to various musculoskeletal diseases for detection [12], classification [13, 14], and segmentation [15]. Among these, the segmentation method involves a process that separates digital images into several pixels and designates a specified region as an area of interest based on threshold segmentation, which has been increasingly adopted for disease diagnosis or surgical planning by visualizing lesions [16–21].

In the orthopedic field, particularly for those dealing with soft tissues other than bony structures, the segmentation method is usually based on MRI data, and several studies have reported successful results in the segmentation of the anterior cruciate ligament [22], knee articular cartilage [23], or meniscus [24] using MRI data. Regarding the shoulder joint, a few deep learning studies on RCT have been conducted using many imaging modalities. Kim et al. [25] introduced a deep learning algorithm that can detect significant RCT based on simple shoulder radiographs and showed 78.1% sensitivity and 87.0% specificity. Thao et al. [26] proposed a deep learning method to automatically detect RCT and visualize tear location using ultrasound images; they reported 88.2% accuracy, 93.8% sensitivity, and 83.6% specificity. Similarly, Wang et al. [27] used ultrasound images to detect and visualize RCT lesions and reported 75% accuracy, 89% recall, and 83% precision. Meanwhile there are also MRI-based deep learning studies for rotator cuff. Kim et al. [28] introduced an automatic rotator cuff muscle measuring algorithm and showed that the Dice coefficient of the occupation ratio was 0.94, and the accuracy was 0.99. Similarly, Martin et al. [15] performed automatic rotator cuff muscle segmentation using a deep learning algorithm and reported a 0.93 Dice score. Recently, a study by Yao et al. [29] investigated the automatic segmentation of RCT lesions using MRI data and reported an 81.4% dice segmentation accuracy in 2D MR coronal images.

However, these studies adopted simple radiograph or ultrasound for the detection of RCT and subject of the studies were confined to the rotator cuff muscle, not the torn rotator cuff tendon. To the authors’ knowledge, studies focusing on the torn rotator cuff tendon are lacking and there is no study about RCT segmentation using 3D MRI.

Thus, the purpose of this study was (1) to develop a deep learning technique that can automatically detect and segment RCT lesions using 3D MRI data and to visualize the segmented area in a three-dimensional reconstructed image; (2) to evaluate the accuracy and efficacy of rotator cuff tendon tear segmentation.

## Materials & methods

This is a retrospective study and the study protocol was approved by the Ethics Committee of Konkuk University Medical Center (KUMC 2022-03-041-001) with a waiver of informed consent. MRI data obtained between June 2010 and August 2017 were collected retrospectively, and in-house software was newly developed to carry out the entire process of the study that will be introduced below.

### Data collection

Shoulder MRIs from 303 patients with full-thickness rotator cuff tears (Mean 64.5 ± 8.2 (30–83) years old, 146 men and 157 women) were used as a dataset in this study. The exclusion criteria were as follows: partial thickness tear or intrasubstance tear, isolated subscapularis tear, retear after primary rotator cuff repair, and MRIs in which it is difficult to accurately identify the tear site due to low resolution. Each MRI scan was performed using a 3.0-T system (Signa HDx; GE Healthcare) with a dedicated shoulder coil. The following MRI sequences were used: axial images obtained with T1-weighted spin echo (repetition time/echo time:550-733/15-17 ms) sequences and coronal and sagittal images obtained with T2-weighted spin echo (repetition time/echo time:3500-4000/60-110 ms) sequences. The slice thickness was 4 mm with a slicing gap of 0 or 0.4 mm and a field of view of 16 × 16 cm.

### U-Net model

**Fig 1:**
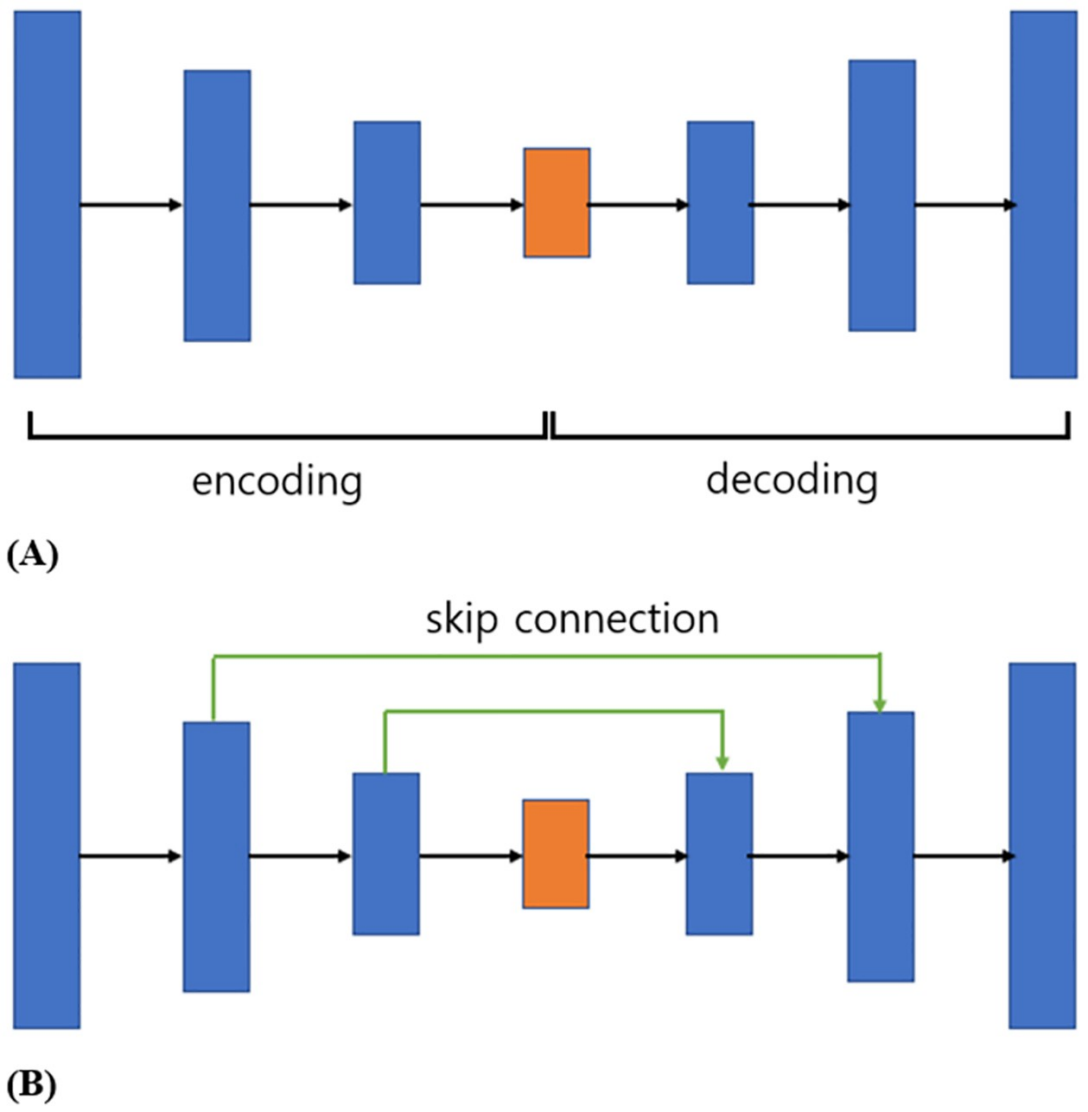
The schematic diagram of U-Net. (A) U-Net encoding-decoding process. In the encoding stage the dimensions are reduced. And in the decoding stage only low-dimensional encoded information is used to reduce the number of channels, and the dimensions increase. (B) U-Net concatenation process. Specific location information is lost during dimensionality reduction in the encoding step, and that loss cannot be recovered in the decoding step. Therefore, a skip connection is used.

For rotator cuff tendon segmentation, the U-Net model proposed by Ronneberger et al. [30] was used for the training. The U-Net model is an encoder-decoder model designed based on the fully convolutional network (FCN) of the end-to-end method, proposed for image segmentation of medical images. The end-to-end learning method involves relocating the multiple steps necessary to solve a problem through a single neural network. Because it is effective when the data to be managed are large, it is known to be suitable for medical data processing, such as in the case of MRI images with dozens of slices rather than a few photo images [30]. The FCN is a model proposed for the semantic segmentation problem and a modified convolutional neural network (CNN) model that has shown excellent performance in related fields. In brief, the U-Net CNN process is as follows. In the encoding stage, the dimensions are reduced while increasing the number of channels to capture the features of the input image. In the decoding stage, only low-dimensional encoded information is used to reduce the number of channels and the dimensions increase to restore the high-dimensional image (Fig 1A). However, detailed location information regarding the image object is lost during dimensionality reduction in the encoding step, and the loss of location information cannot be recovered because only low-dimensional information is used in the decoding step. The basic idea of U-Net is to use low-and high-dimensional information to extract image features and enable accurate location identification. Therefore, a method for concatenating the features obtained from each layer in the encoding step for each layer in the decoding step is used. A direct connection between the encoder and decoder layers is called a skip connection (Fig 1B).

### Preprocessing and data labeling

Preprocessing and labeling of 303 MRI data were performed. Data preprocessing is the process of selecting, using, and transforming data types. The MRI T2 coronal image, in which the RCT lesion was visible, was converted into Nifti image format. Conversion to the Nifti image format was performed using in-house developed software (Reconeasy 3D program, SeeAnn Solution, South Korea, official code: https://github.com/youngjin0011/APShoulder). A generative adversarial network (GAN) algorithm that augments the number of training data with image flip, rotation, movement, and Gaussian noise was applied to increase the training data while preventing overfitting [31, 32]. For the data labeling, margin of the RCT area was outlined manually through the entire MR image using the same in-house developed software by two orthopedic shoulder specialists (S.H.L and J.H.L), and 303 labeled MRI was used for each step of the deep learning (training, validation and testing). If the exact location of the RCT lesion was ambiguous, it was re-evaluated and confirmed after a thorough discussion with an expert group consisting of two senior shoulder specialists (S.W.C. and J.P.Y.) and one musculoskeletal radiologist (S.G.M.).

### U-Net learning process

A brief description of the U-Net architecture (official code: https://github.com/youngjin0011/APShoulder) is as follows: When using DICOM data composed of 100 slides of 512 × 512 pixels, the entire image data becomes a 512 × 512 × 100 image block with volume, and each pixel is called a voxel, compound word of volume, and pixel. Labeled data is an image block in which the RCT region is set to 1, the rest of the region is set to 0, and the size is 512 × 512 × 100, which is the same size as the original DICOM data. In U-Net learning, this DICOM-labeled data pair is input as a question paper and a correct answer paper. After passing through the U-Net learning model using the input data, the DICOM and labeled data were inferred to generate a weighted data block for each voxel. This weighted block has a value corresponding to each voxel and has a value close to 0 in the case of a region to be excluded and close to 1 in the case of a region to be included. Through this process, for all given training data pairs, inferences are gathered by accumulating weighted data blocks. After learning all the given training data once, using the validation data (the validation dataset is a data sample that is used as a reference to measure the learning state, but is not used for training and final evaluation), 512 × 512 × 100 Nifti image data are generated, which is the result of finding the RCT lesion. The current learning accuracy is calculated by comparing each voxel of the labeling data of the validation data with the Nifti data, which is the result of guessing the RCT lesion.


$$\frac{number\;of\;matching\;voxels\;}{total\;number\;of\;voxels}=accuracy$$

**Fig 2:**
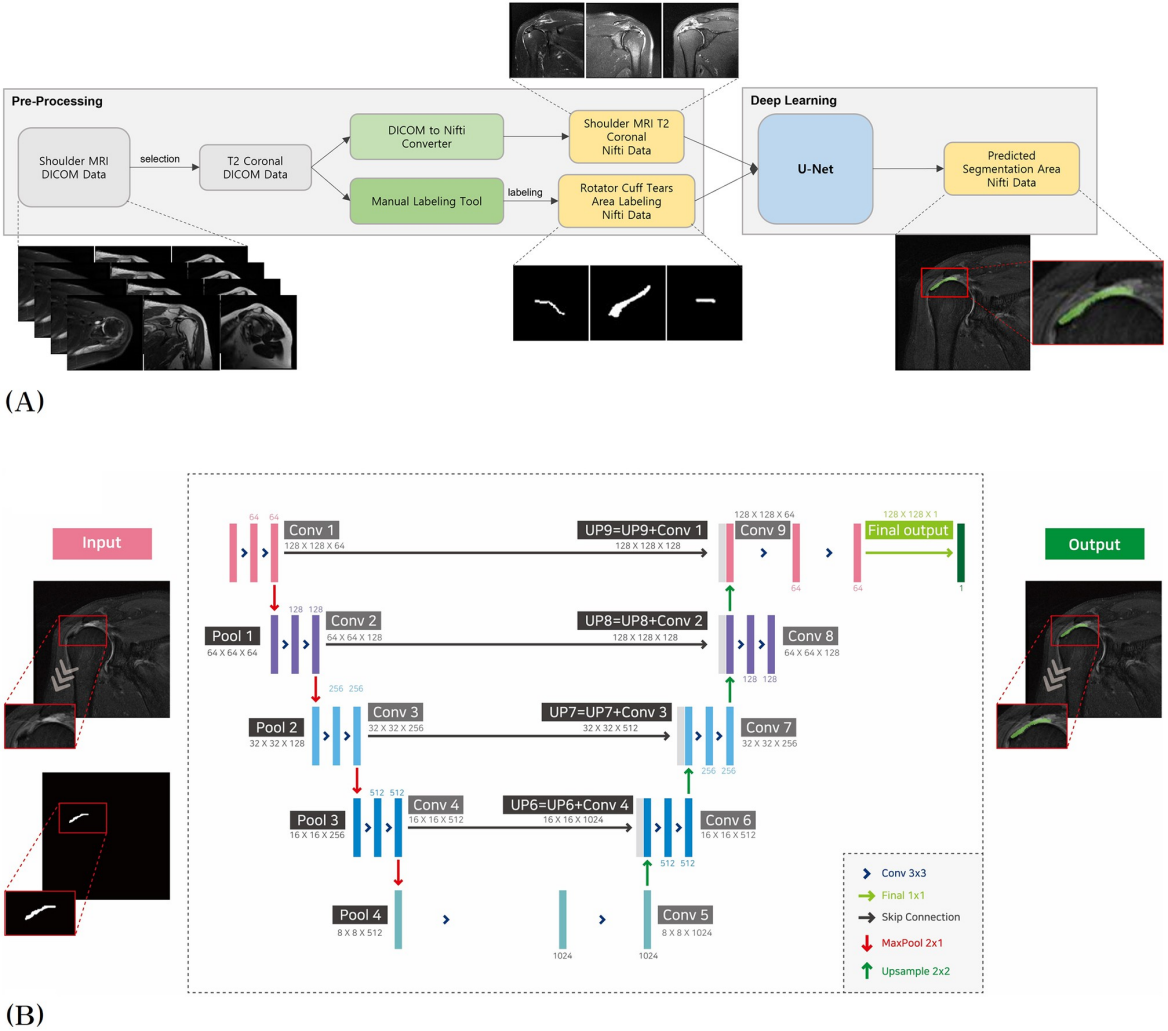
Entire architecture of 3D U-Net for the RCT segmentation. (A) Flowchart of the preprocessing and U-Net learning. The software selects T2 coronal data to be used for learning and loads labeling data. Both training and labeling data are converted into the Nifti image format. After learning with U-Net model, the result of prediction is generated in Nifti format. (B) Structure of U-Net model. The input data goes through the contracting path on the left and the expending path on the right. Both pathways are symmetrical to each other. Finally, it creates a segmentation map that classifies each pixel in the image.

This process was repeated many times, and the quality of the learning result was calculated using the test data. When the final accuracy was greater than or equal to the target accuracy, the weighted data of the learning result were extracted. Finally, if the learning results are applied to a new MRI DICOM image of the shoulder joint, the result of automatic image segmentation of the rotator cuff tear region can be obtained with a high probability. In this study, training, validation, and testing were performed using 303 MRI data, and the data were randomly allocated by the software for training, validation, and testing at a ratio of 6:2:2. The U-Net process for automatic image segmentation of the RCT legion consists of data preprocessing and deep learning, as shown in Fig 2.

### Evaluation of the model performance

The performance of the learning model for RCT lesion segmentation was evaluated by measuring the values of sensitivity, specificity, Dice coefficient, precision, F1-score, and Youden index. The calculation formulae are as follows.


$$Sensitivity=\frac{TP}{TP+FN}\times 100$$

$$Specificity=\frac{TN}{FP+TN}\times 100$$

$$Dice=\frac{2\times TP}{\left(TP+FP)+(TP+\;FN\right)}\times 100$$

$$Precision=\frac{TP}{TP+FP}\times 100$$

$$F1\;score=\frac{1}{\frac{1}{precision}+\frac{1}{recall}}\times 100$$

Youdenindex=sensitivity+(specificity-1)

The area segmented by the learning model is compared to labeled data in units of voxels, and when it is calculated as true or false otherwise, TP is true positives, FP is false positives, TN is true negatives, and FN is false negatives. TP is when the area to be found is well-found, FP is when the area to be found is not found, TN is when the area to be excluded is excluded, and FN is when the area to be excluded is included.

### Statistical analysis

All statistical analyses were performed using SPSS 17.0 (SPSS, Inc., Chicago, IL, USA). Descriptive statistics were used to report each value of the Dice coefficient, sensitivity, specificity, precision, F1-score, and Youden index, which are described as median and interquartile range. The Dice coefficient was used to assess the segmentation accuracy. Sensitivity was the ratio of finding the tear region well, specificity was the ratio excluding the non-tear region well, and Dice was the difference between the correct answer and the predicted value. The probability threshold for a positive finding was determined using Youden’s index.

## Results

### Visualization of the rotator cuff tear region

**Fig 3:**
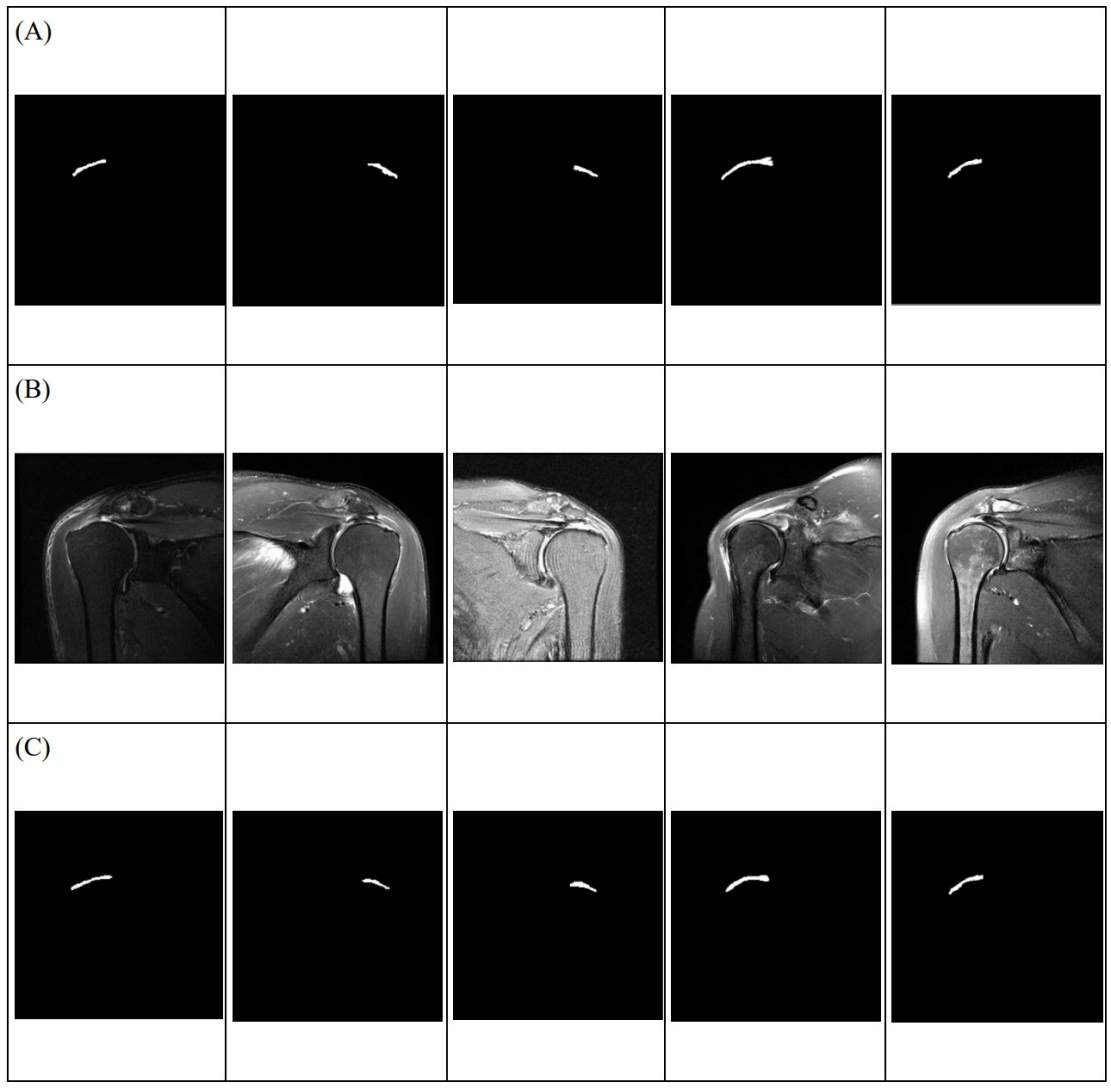
Segmented image of the rotator cuff tear region. Each row is (A) ground truth based on manual labeling data by shoulder specialists, (B) raw MRI data, and (C) automatic segmentation results by the proposed learning model.

**Fig 4:**
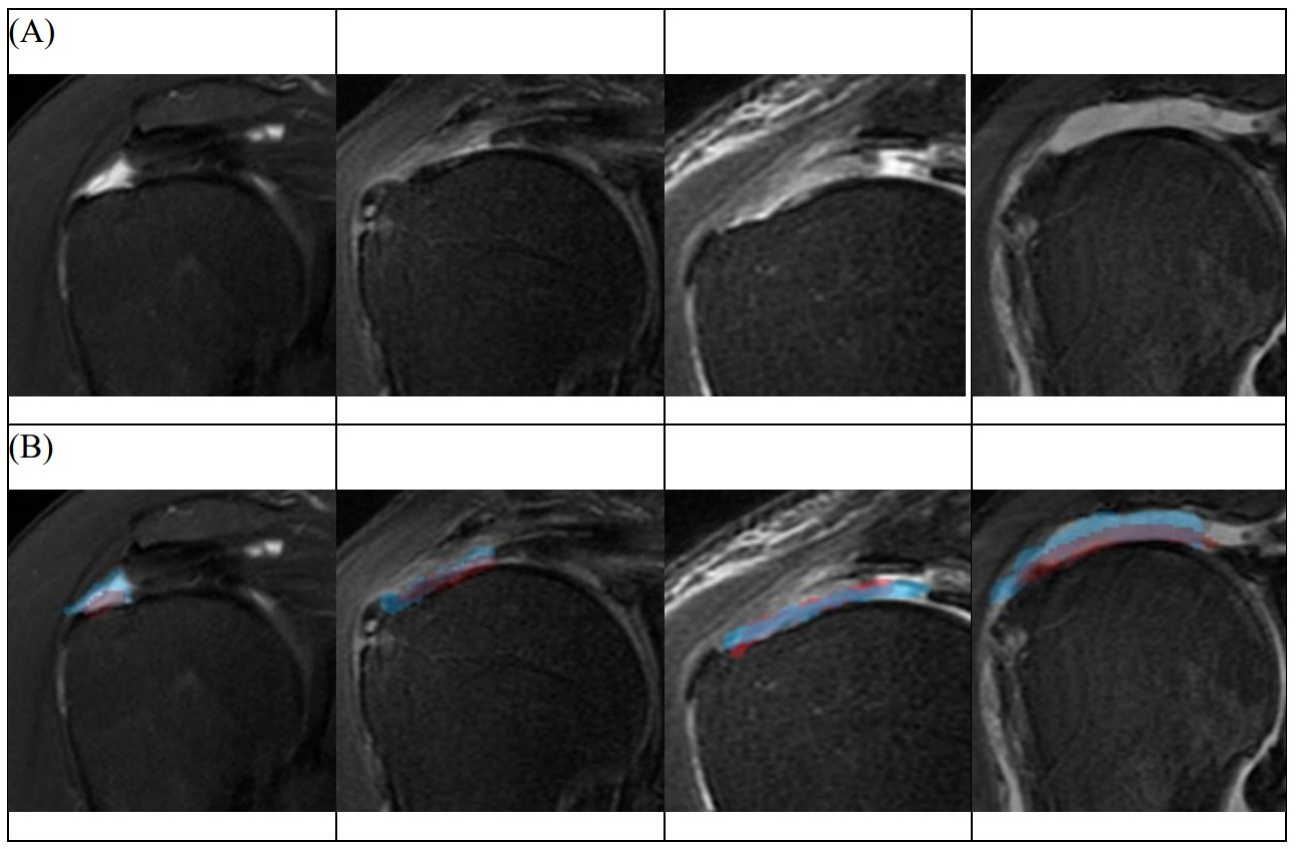
Results of segmentation corresponding to rotator cuff tear site. (A) Examples of the original MRI image which show rotator cuff tear. (B) The red area indicates the manually labeled region by shoulder specialists, and the blue area indicates the segmented region by the proposed learning model.

An automatically segmented RCT lesion was successfully demonstrated by applying the proposed learning model. Examples of manual labeling and automatic segmentation are shown in Figs 3 and 4, respectively.

**Fig 5:**
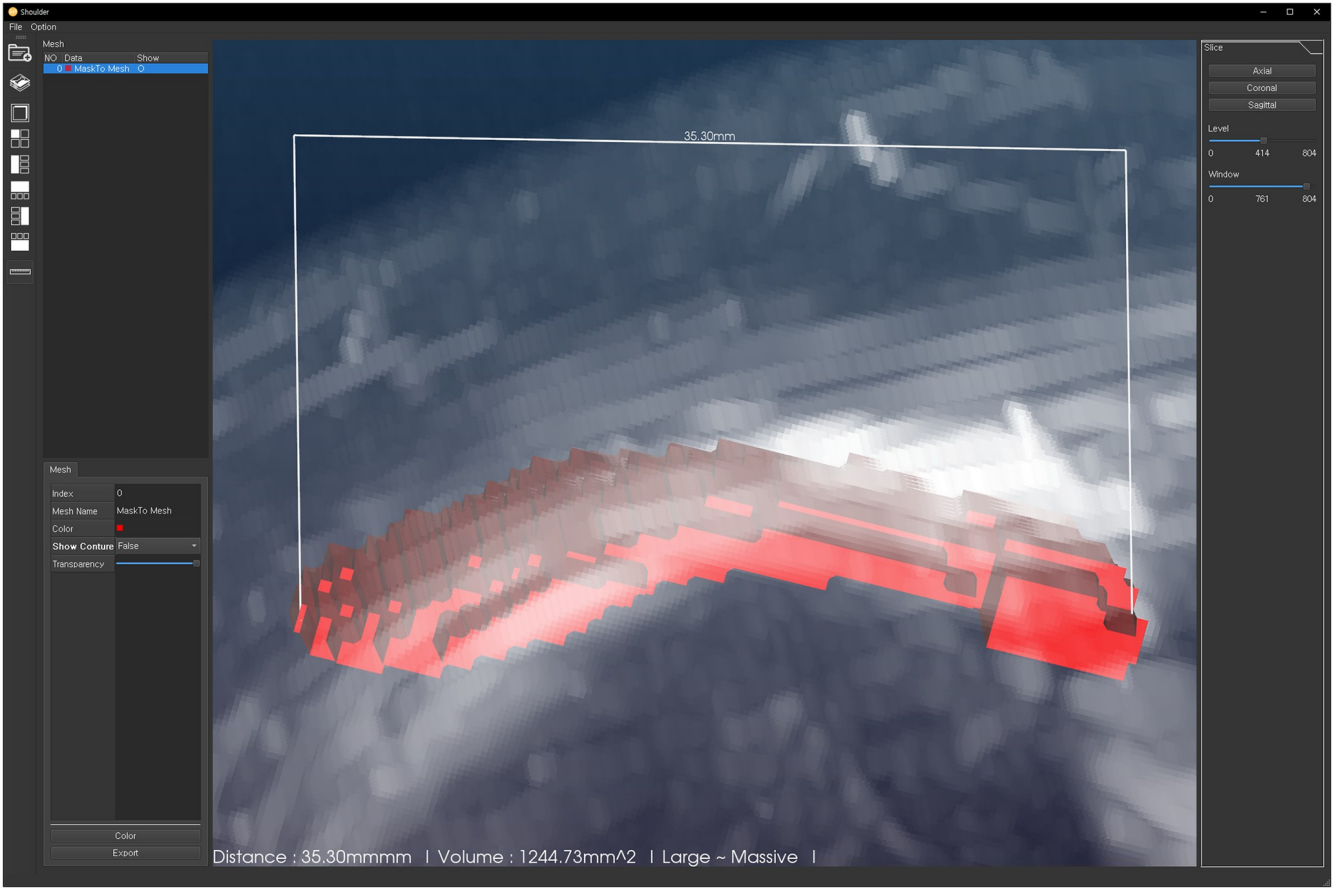
Three-dimensional visualization of the rotator cuff tear region. The final segmented image was converted into three-dimensional reconstructed image using in-house software. The maximal distance of tear was automatically measured and presented at the bottom of the image (in white letters) together with the tear classification.

The proposed model also successfully visualized the tear region in 3D MRI, with further automatic calculation of the maximal distance and volume of the tear region and displaying the tear size category (small/medium/large/massive) (Fig 5).

**Fig 6:**
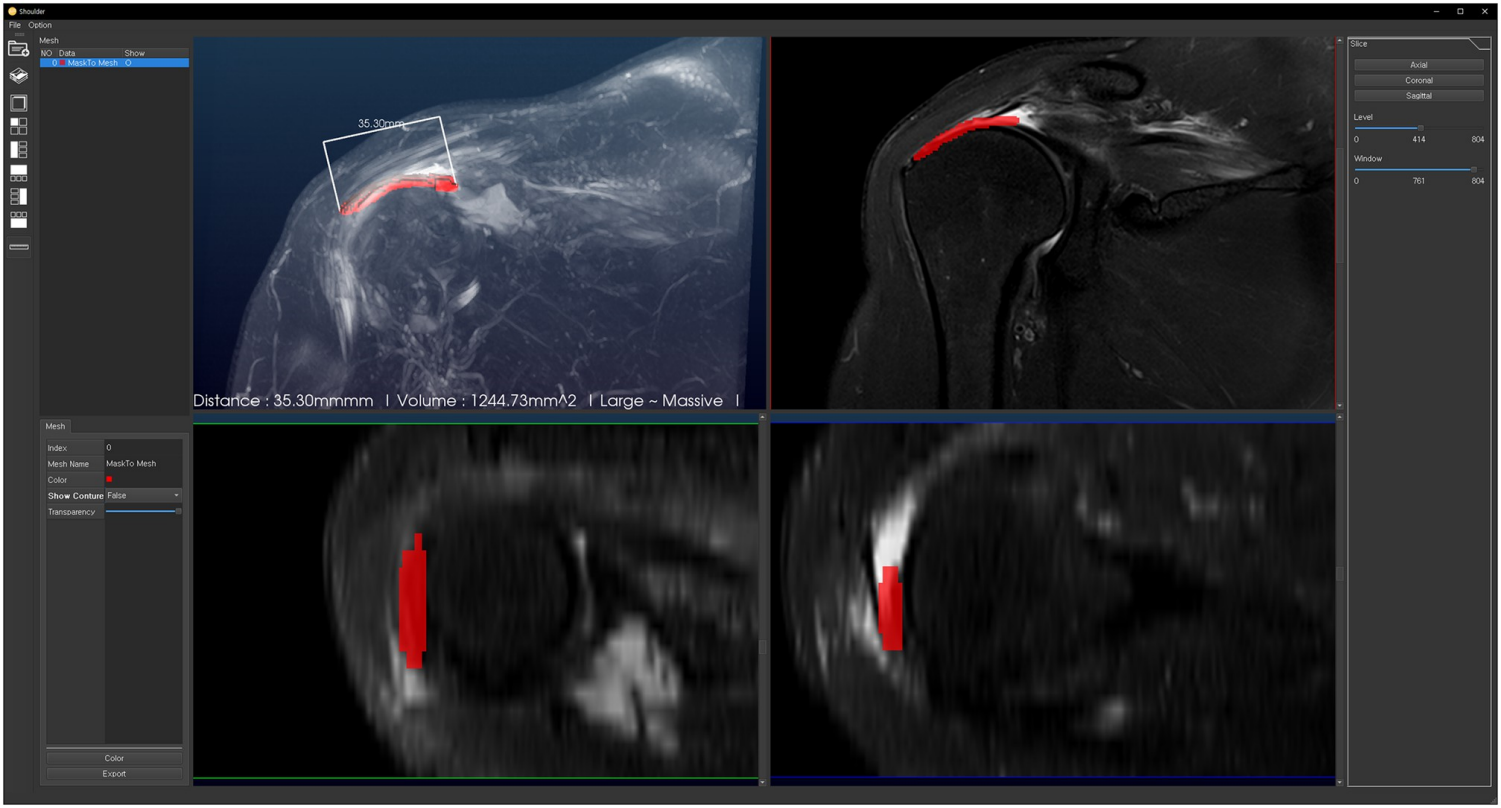
Multiplanar reformation view. It shows automatically segmented RCT lesion (red area). Segmented area can be viewed two dimensionally in multiplanar (coronal, axial, sagittal) direction and controlled freely.

In addition to the 3D image, it supplied the segmented 2D RCT lesion in a multi-planar reformation view to help the user obtain a better spatial understanding of the structure in each coronal, axial, and sagittal image (Fig 6). In program when the raw MRI data is loaded, the ruptured site is automatically found within a few seconds. Segmented image can be showed and controlled freely on axial, sagittal and coronal view (S1 Video).

### Performance of the proposed model

**Fig 7:**
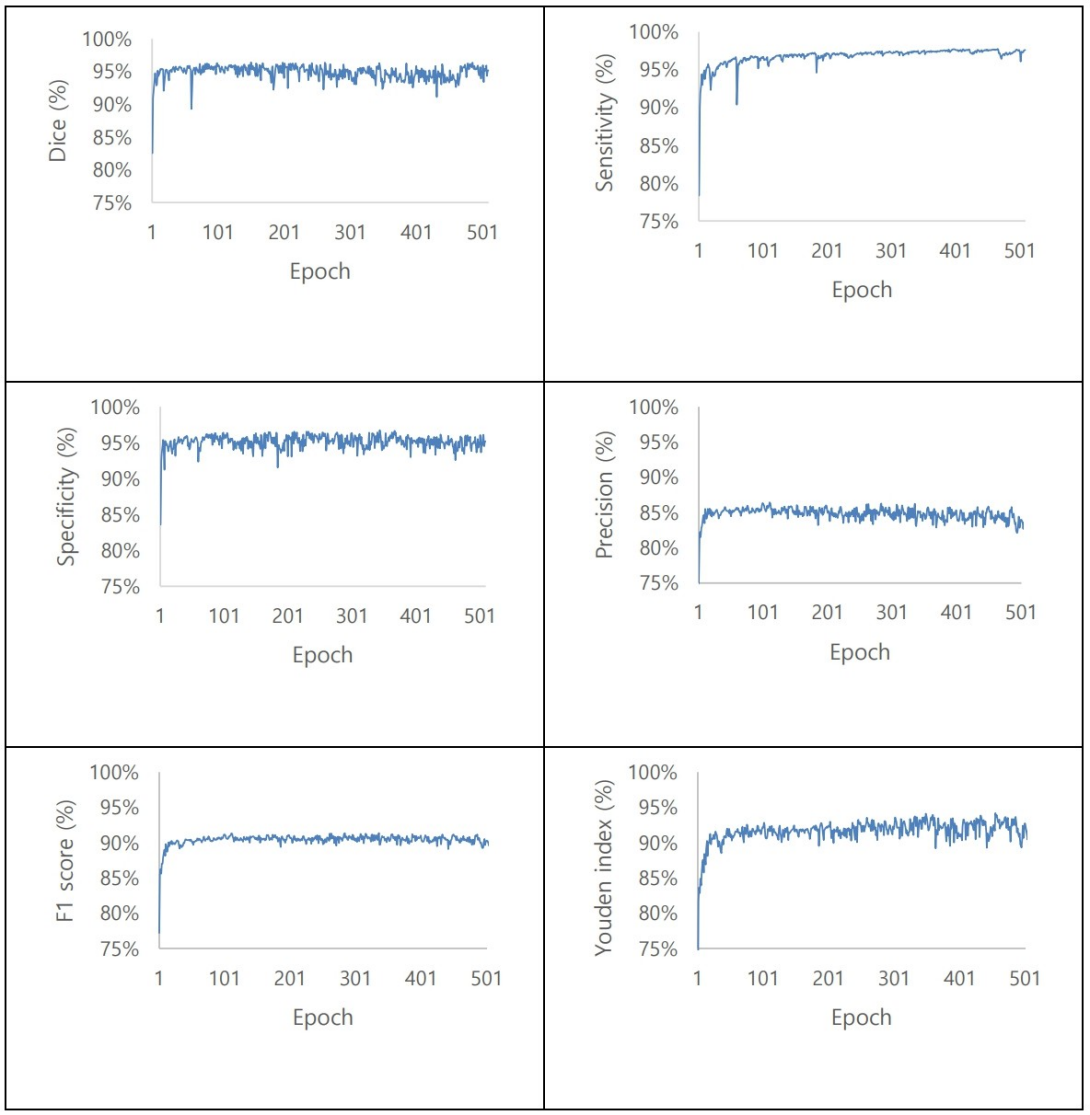
Performance of the rotator cuff tear segmentation. Dice, sensitivity, specificity, precision, F1 score, and Youden index.

Approximately 500 repeated learning sessions were conducted to obtain the best results. Consequently, the Dice score had an accuracy of 94.3%. The sensitivity was 97.1%, specificity was 95.0%, precision was 84.9%, F1 score was 90.5%, and Youden index was 91.8% (Fig 7 and Table 1).

**Table 1:** Performance of the rotator cuff tear segmentation. Values are presented as the median and interquartile range (Q1–Q3).

Variable	Value (%)
Dice	94.3 (93.7, 94.8)
Sensitivity	97.1 (96.6, 97.4)
Specificity	95.0 (94.4, 95.5)
Precision	84.9 (84.4, 85.4)
F1 score	90.5 (90.2, 90.8)
Youden index	91.8 (91.2, 92.5)

## Discussion

This study showed that the proposed deep learning model for RCT segmentation provided satisfactory outcomes with a high Dice score accuracy of 94.3%. In addition, the software successfully visualized RCT lesions through three-dimensional reconstructed image and provided information about tear shape and size. Although a segmentation study for the RCT lesion using MRI data is scarce, several previous studies that have dealt with similar soft tissue lesions, such as knee anterior cruciate ligament (84%) [22], intervertebral disc (89%) [33], or knee meniscus (86.4%) [24], have reported approximately 80–90% of Dice scores for each relevant soft tissue lesion segmentation. Compared to these results, the accuracy of RCT segmentation of our study seems to be quite high. Furthermore, accurate 3D segmentation of RCT lesions may help us to understand tear patterns and sizes more intuitively and precisely.

Several deep learning studies have tried to segment RCT lesion using various modalities. For example, Kim et al. [25] utilized simple radiograph, but their study had an inherent limitation of using shoulder radiographs. Namely, it is not a confirmative diagnostic modality for RCT and also cannot be used to show the RCT lesion directly [34, 35]. In addition, although the detection based on simple radiographs may be meaningful as a screening test, we worry about that the sensitivity and specificity are unsatisfactory in clinical practice. Thao et al. [26] and Wang et al. [27] used ultrasound images. However, the accuracy is still low compared to our MRI-based method. And the need for a skillful sonographer, reproducibility, and artifacts of ultrasound [36] can be pointed out as limitations that are hard to ignore. MRI-based deep learning research studied by Kim et al. [28] is meaningful in that they demonstrated highly accurate segmentation results using MRI data; however, they focused on rotator cuff muscles atrophy in a 2D MRI image and not the rotator cuff tendon where the tear occurs. Thus, in our previous study, we developed a deep learning algorithm for the detection and classification of RCT using MRI data, and it showed an accuracy of 92.5% for RCT detection and 76.5% for RCT classification [37]. However, although the RCT lesion could be localized using a class activation map, the segmentation of the RCT lesion were not performed. Therefore, as an extension of the previous study, we sought to develop an accurate and efficient deep learning technique for the segmentation of the RCT lesion, and successfully segmented and visualized the RCT lesion with a 94.3% Dice score in this study. Recently, Yao et al. [29] focused on RCT area and segmented it with satisfactory results. But unlike our 3D MRI, they used 2D MR coronal image. 3D MRI can provide more enriched information about the configuration of the tear by visualizing the RCT lesion in 3D space rather than 2D space. Indeed, it may be difficult to get the whole picture of tear pattern just at the glance of a slice of 2D MR images. Especially, it will be even more challenging if the tear pattern is very complex and there is a lack of specialized knowledge about the shoulder anatomy.

We anticipate that our segmentation and visualization method may be of great help. For example, it can be helpful when a surgeon establishes a surgical plan or when explaining to a patient about the surgery. It also may be useful when communicating or discussing between doctors about the lesions. Furthermore, our developed software can segment and automatically measure the maximum length of the tear in 3D and classify the RCT lesion according to the tear size, ranging from small to massive tears. Therefore, we believe it may be potentially valuable for many medical personnel to read and interpret complex RCT lesions in shoulder MRIs, particularly those who are not experienced in orthopedic surgery.

MRI is an essential tool for RCT diagnosis; however, general orthopedic surgeons and even shoulder specialists require much times to identify an RCT lesion because dozens of axial, sagittal, and coronal MRI images should be carefully reviewed. Additionally, more time is required to review RCTs with complex tear patterns. In order to more easily identify complex RCT patterns, Gyftopoulos et al. has conducted the study about three-dimensional magnetic resonance reconstructions [38]. They emphasized on the fact that the overall accuracy of 3D MRI was higher from 2D MRI accuracy. However, margins of the rotator cuff had to be outlined manually one by one to obtain 3D reconstruction image. On the contrary, our proposed deep learning model takes only a few seconds to segment RCT lesions and visualize them in a three-dimensional reconstruction image. Considering the time required to read and consistency with less bias, this deep learning-based segmentation of RCT lesions is more encouraging. Furthermore, the socioeconomic burdens for the repeated and professional MRI reading for RCT may be partially solved using this deep learning algorithm. But probably more studies will be needed to improve the performance and efficiency of the deep learning algorithm and to apply it to clinical practice. Nevertheless we believe that this proposed deep learning model can act as an alternative to solve the problem of the time-consuming process of MRI reading for RCT reliably and efficiently [39]. And it may help clinicians make decisions for optimal treatment of RCT and communicate with patients.

**Fig 8:**
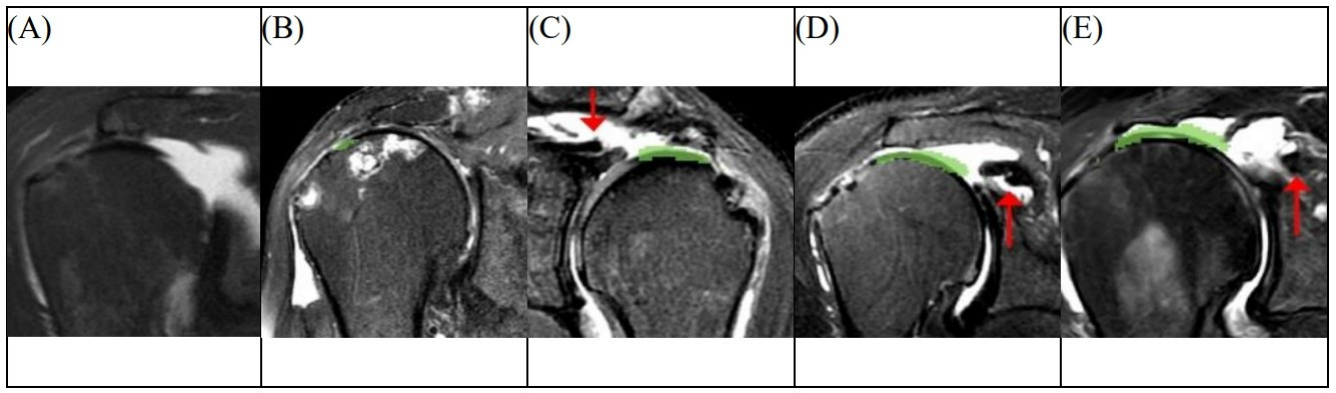
Examples of incorrectly segmented cases. In some cases of massive tears with cuff tear arthropathy, the result was interpreted as if there was no tear or a small-sized tear (A, B). In some cases of delamination tear patterns, the algorithm only recognized less retracted tear portions without detecting the more retracted medial ends (the red arrow) (C, D, and E).

This was the first study to automatically segment RCT lesions of 3D MRI using a deep learning model with high accuracy. Nevertheless, this study has several limitations. First, this study only included posterosuperior cuff tears and did not include subscapularis tears. As full-thickness subscapularis tears are extremely rare, it was not possible to gather sufficient MRI data for the analysis and segmentation of the subscapularis tear [40]. However, for a more thorough diagnosis of RCT, additional follow-up studies including enough MRI data of the subscapularis tears with various tear sizes are needed as the next step of the study. Second, the number of MRI scans included in this study was relatively small. Although we confirmed the considerably high accuracy of the segmentation in our MRI dataset using the proposed learning model, a larger amount of MRI data may be beneficial in increasing the accuracy and minimizing any technical problems, such as overfitting. Third, there is a limitation in the detection and segmentation of RCT cases with unusual configurations. For example, in some cases of massive RCT with cuff tear arthropathy, the software could not detect the tear site. This may be due to the superior migration of the humeral head. The elevated humeral head masked the RCT and the result was interpreted as if there was no tear. In some cases of delaminated tears, the algorithm did not detect the more retracted tear portion and only recognized a less retracted tear portion (Fig 8). In such exceptional cases, professional interpretation and analysis by a human reader may still be required, and a more sophisticated algorithm with much more data including many exceptional cases may be required.

## Conclusion

The proposed deep learning model for the 3D segmentation of RCT lesions showed an overall high accuracy and it demonstrated successful 3D visualization of segmented area. This in-house software also offers maximum length of the tear in 3D and classifies the RCT lesion automatically just in a few seconds. More studies will be needed to improve the performance and efficiency of the deep learning algorithm and to determine the feasibility of its clinical application. Nevertheless, we believe that this proposed deep learning model can act as an alternative to solve the problem of the time-consuming process of MRI reading for RCT reliably and efficiently. And it also may help clinicians make decisions for effective treatment of RCT and communicate with patients.

## Supporting information

S1 Video Whole process of running in-house software. (MP4)

S1 Data Minimal data set. (XLSX)
